# Supplementary material for: Long-term follow-up of mandibular dental arch changes in patients with complete non-syndromic unilateral cleft lip, alveolus, and palate
Source: PeerJ. 2021 Dec 16;9:e12643. doi: 10.7717/peerj.12643 (PMC8684719; doi:10.7717/peerj.12643)
Supplement: Supplemental Information 1 — Intra-observer reliability and measurement error for the mandibular intercanine (3-3,) inter first premolar (4-4), inter second premolar (5-5), inter first molar (6-6), inter second molar (7-7) distances at 3 different time points. Reliability expressed by Pearson’s correlation coefficient. DME = duplicate measurement error (in mm); Mean diff = mean difference between first and second measurement and 95% confidence interval (in mm). Results of paired t-test for the mean diff (p-values). [file peerj-09-12643-s001.docx]

**Supplementary Table 1** Intra-observer reliability and measurement error for the mandibular intercanine (3-3,) inter first premolar (4-4), inter second premolar (5-5), inter first molar (6-6), inter second molar (7-7) distances at 3 different time points. Reliability expressed by Pearson’s correlation coefficient. DME = duplicate measurement error (in mm); Mean diff = mean difference between first and second measurement and 95% confidence interval (in mm). Results of paired t-test for the mean diff (p-values).

| **Distance** | **Time** | **N** | **Reliability** | **DME** | **Mean diff** | **95% CI of diff** | **P mean diff** |
| --- | --- | --- | --- | --- | --- | --- | --- |
| 3-3 | T0 | 20 | 0.970 | 0.26 | -0.12 | [-0.29...0.06] | 0.181 |
|  | T2 | 17 | 0.932 | 0.46 | 0.01 | [-0.32...0.34] | 0.946 |
|  | T5 | 13 | 0.991 | 0.19 | 0.07 | [-0.09...0.23] | 0.385 |
| 4-4 | T0 | 20 | 0.955 | 0.39 | 0.003 | [-0.25...0.26] | 0.981 |
|  | T2 | 17 | 0.939 | 0.54 | -0.20 | [-0.60...0.19] | 0.295 |
|  | T5 | 13 | 0.984 | 0.30 | -0.11 | [-0.37...0.15] | 0.387 |
| 5-5 | T0 | 20 | 0.986 | 0.33 | -0.11 | [-0.33...0.11] | 0.319 |
|  | T2 | 17 | 0.990 | 0.37 | -0.01 | [-0.27...0.26] | 0.958 |
|  | T5 | 13 | 0.994 | 0.26 | -0.12 | [-0.34...0.11] | 0.279 |
| 6-6 | T0 | 20 | 0.996 | 0.23 | 0.005 | [-0.15...0.16] | 0.949 |
|  | T2 | 17 | 0.993 | 0.30 | -0.04 | [-0.27...0.17] | 0.668 |
|  | T5 | 13 | 0.993 | 0.35 | -0.04 | [-0.34...0.25] | 0.752 |
| 7-7 | T0 | 20 | 0.985 | 0.36 | -0.17 | [-0.41...0.06] | 0.146 |
|  | T2 | 17 | 0.985 | 0.39 | 0.03 | [-0.25...0.31] | 0.837 |
|  | T5 | 13 | 0.995 | 0.25 | -0.05 | [-0.26...0.16] | 0.630 |
