# Supplementary figures and images for: Long-term follow-up of mandibular dental arch changes in patients with complete non-syndromic unilateral cleft lip, alveolus, and palate
Source: PeerJ. 2021 Dec 16;9:e12643. doi: 10.7717/peerj.12643 (PMC8684719; doi:10.7717/peerj.12643)

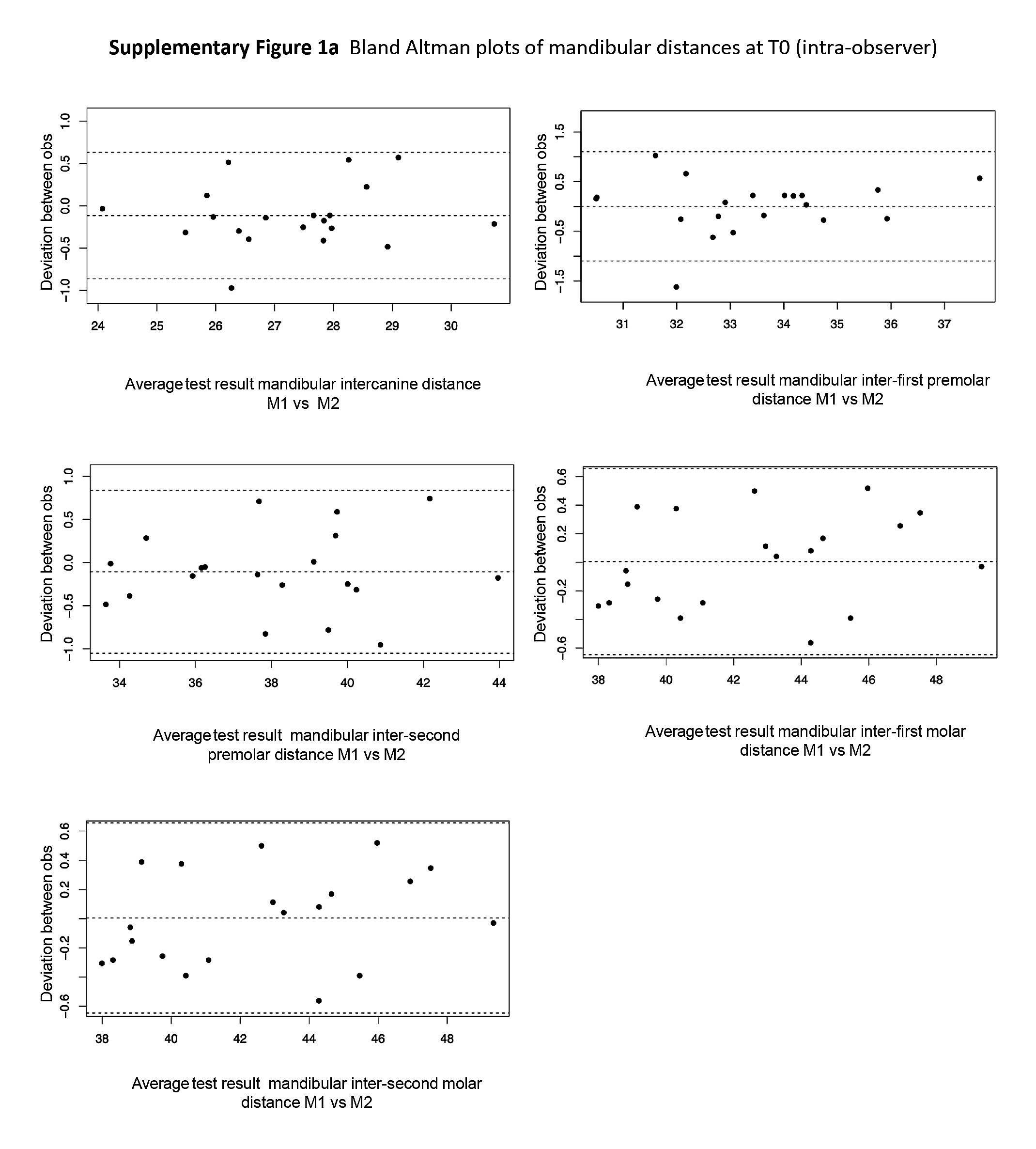

Supplement: Supplemental Information 2 — Bland Altman plots of mandibular distances at the end of treatment (intra-observer). [file peerj-09-12643-s002.png]

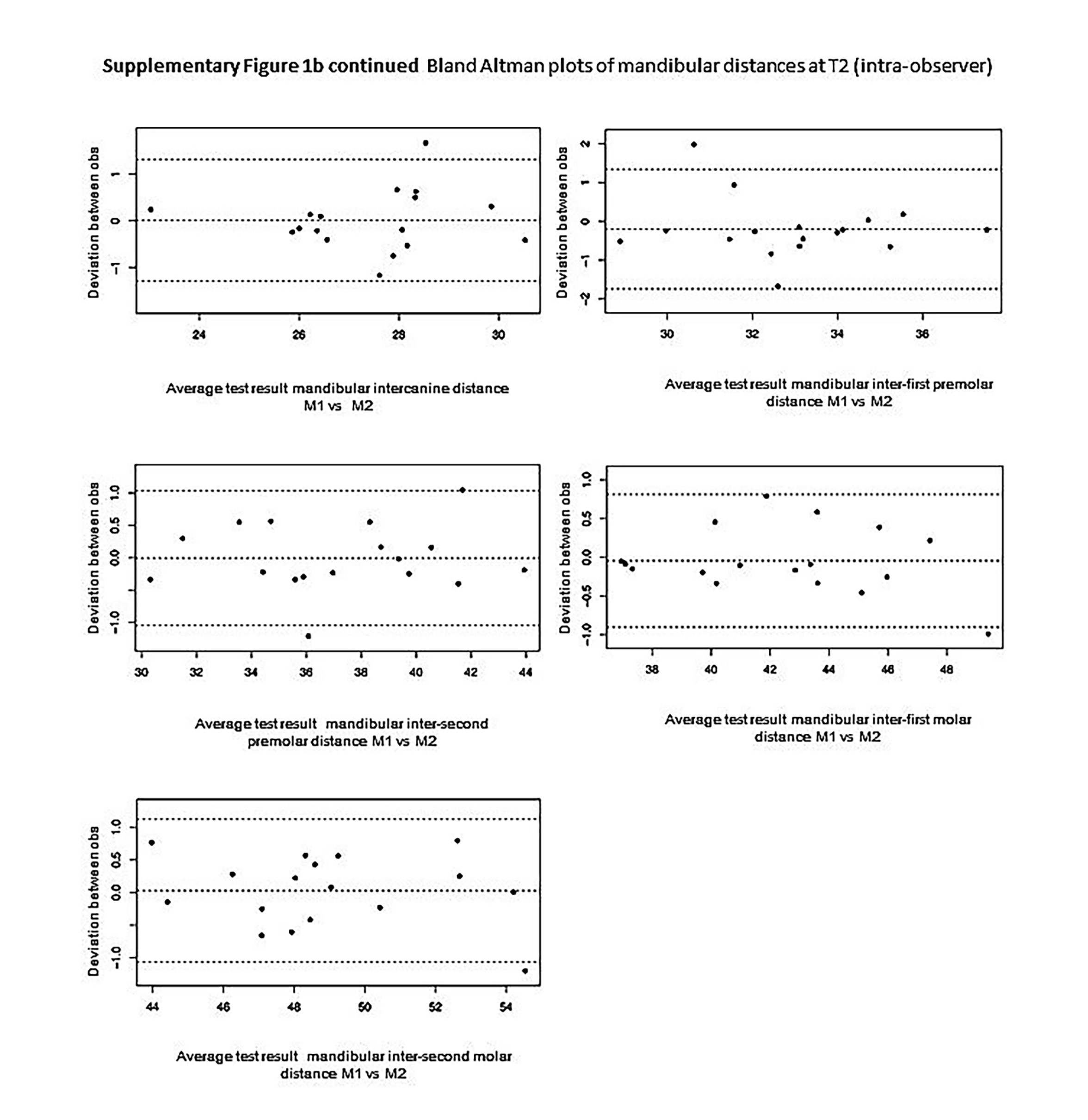

Supplement: Supplemental Information 3 — Bland Altman plots of mandibular distances at 2 years after treatment (intra-observer). [file peerj-09-12643-s003.png]

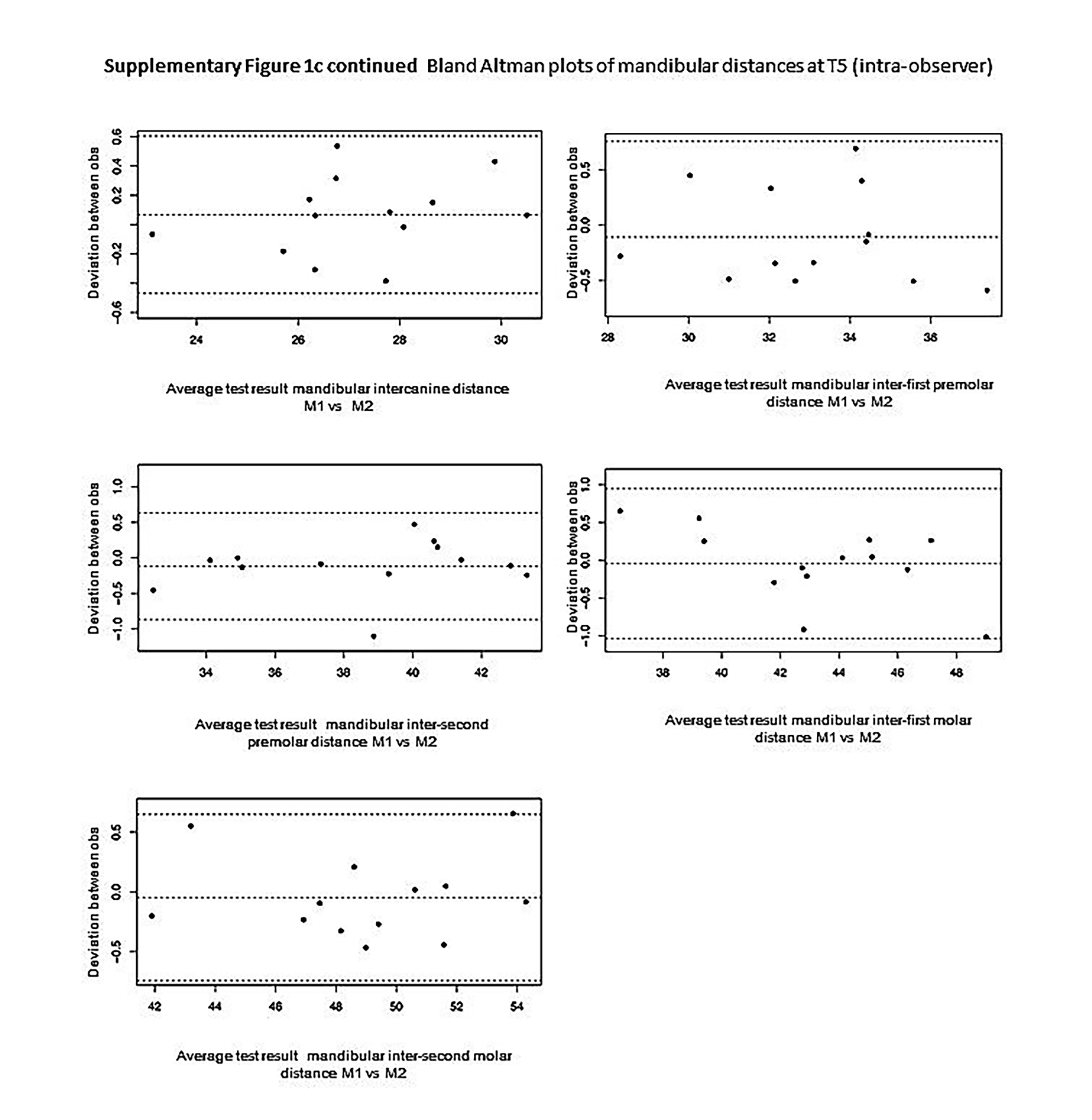

Supplement: Supplemental Information 4 — Bland Altman plots of mandibular distances at 5 years after treatment (intra-observer). [file peerj-09-12643-s004.png]

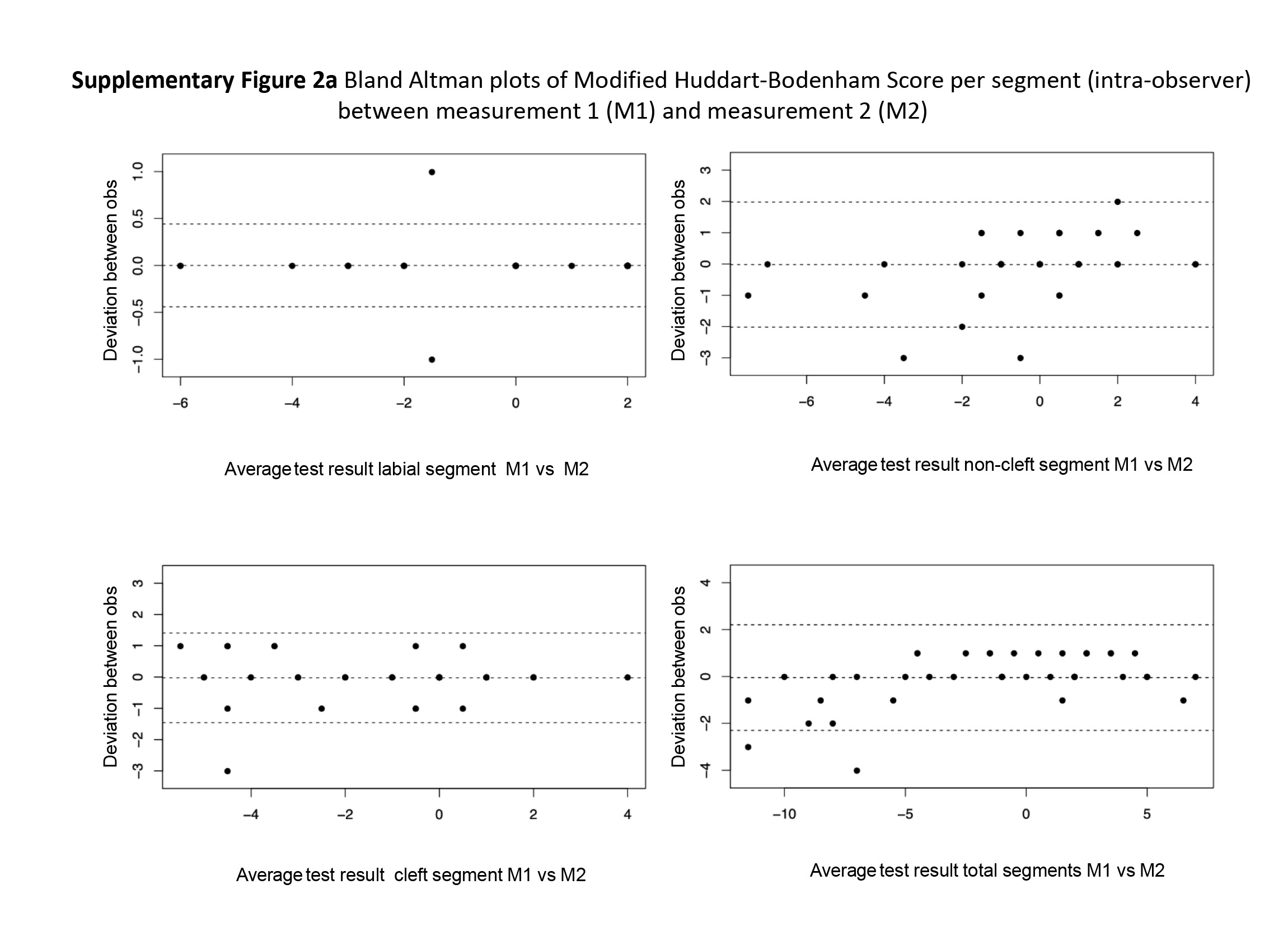

Supplement: Supplemental Information 5 — Bland Altman plots of the Modified Huddart-Bodenham scores (intra-observer) between measurement 1 (M1) and measurement 2 (M2) for the labial segment, buccal cleft and non-cleft segment, and total arch constriction. [file peerj-09-12643-s005.png]

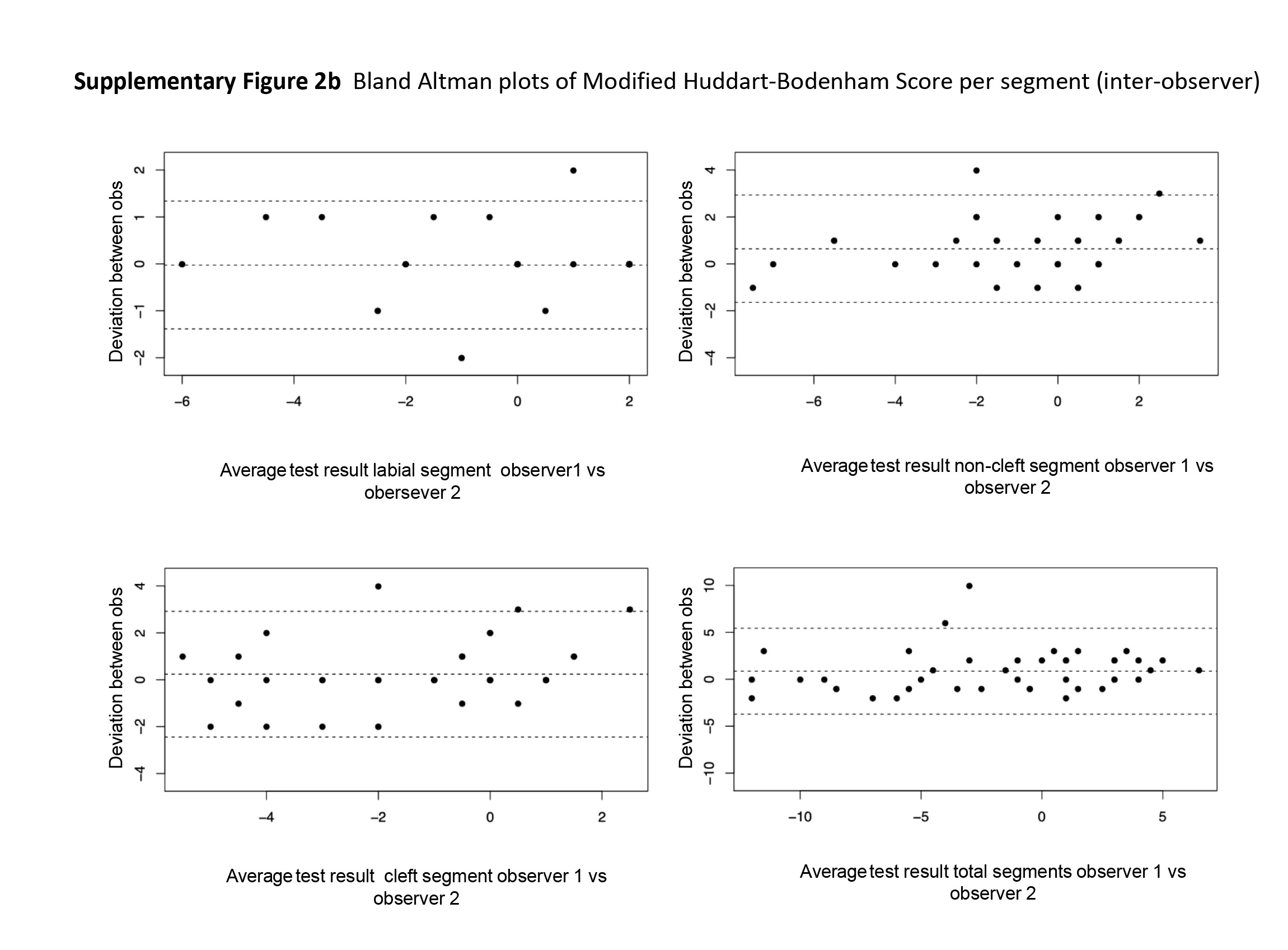

Supplement: Supplemental Information 6 — Bland Altman plots of the Modified Huddart-Bodenham score (inter-observer) for the labial segment, buccal cleft and non-cleft segment, and for total arch constriction. [file peerj-09-12643-s006.png]
